# Biological Aging and Venous Thromboembolism: A Review of Telomeres and Beyond

**DOI:** 10.3390/biomedicines13010015

**Published:** 2024-12-25

**Authors:** Rafaela Vostatek, Cihan Ay

**Affiliations:** Division of Haematology and Haemostaseology, Department of Medicine I, Medical University of Vienna, 1090 Vienna, Austria; rafaela.vostatek@meduniwien.ac.at

**Keywords:** biological aging, venous thromboembolism, telomeres

## Abstract

Although venous thromboembolism (VTE) is the third most common cardiovascular disease, and the risk of VTE increases sharply with advancing age, approximately 40% of VTE cases are currently classified as unprovoked, highlighting the importance of risk factor research. While chronological aging is associated with the risk of VTE, the association with biological aging remains unclear. Biological aging is highly complex, influenced by several dysregulated cellular and biochemical mechanisms. In the last decade, advancements in omics methodologies provided insights into the molecular complexity of biological aging. Techniques such as high-throughput genomics, epigenomics, transcriptomics, proteomics, and metabolomics analyses identified and quantified numerous epigenetic markers, transcripts, proteins, and metabolites. These methods have also revealed the molecular alterations organisms undergo as they age. Despite the progress, there is still a lack of consensus regarding the methods for assessing and validating these biomarkers, and their application lacks standardization. This review gives an overview of biomarkers of biological aging, including telomere length, and their potential role for VTE. Furthermore, we critically examine the advantages and disadvantages of the proposed methods and discuss possible future directions for investigating biological aging in VTE.

## 1. Introduction

Venous thromboembolism (VTE) manifests as deep vein thrombosis (DVT) and pulmonary embolism (PE) and is the third most frequent cardiovascular disease (CVD) after ischemic heart disease and stroke [1,2]. DVT occurs when a blood clot is formed in deep veins, usually of the legs, but also of arms or groin, while PE manifests when a blood clot breaks free from the vein wall and embolizes to the arteries of the lung, blocking the blood supply, which can be potentially life-threatening [3]. There are about 10 million VTE cases annually worldwide, and based on epidemiological modeling in Europe, about 370,000 VTE cases are fatal, classifying VTE as a major clinical health burden [3,4]. It affects all ethnicities, genders, and age groups, albeit the risk of VTE increases sharply with advancing age. The incidence of VTE starts to rise above the age of 55 years and is two to seven times higher compared to a younger cohort aged 40 years or younger [5,6].

While the association of chronological aging with the risk of VTE is established, its association with biological aging remains unclear. In fact, biological aging involves a series of processes leading to the gradual decline of organ viability over time, whereas chronological aging simply describes the passage of time [7]. As a result, individuals sharing the same chronological age may age differently, influenced by variations in their underlying biological aging mechanisms [8]. Factors like heredity, physical fitness, and external environmental stressors contribute to accelerated biological aging [9]. Biological aging reflects generally the health status and has been related to predicting the onset of age-related disorders, including CVD, diabetes, cancers, and Alzheimer’s disease, and mortality [10,11,12]. One goal of the better understanding and analysis of biological age is aimed at recognizing, delaying, or preventing aging-related diseases and disabilities [13]. In the case of VTE, there are several changes in the coagulation system, which occur with aging [14]. For example, aging influences the plasma levels of coagulation proteins and antifibrinolytic factors or biomarkers reflecting activation of the coagulation system and hypercoagulability. Furthermore, aging leads to an overproduction of reactive oxygen species (ROS). These changes are considered to be physiological during the aging process and result in abnormalities in blood flow, vessel walls, and blood constituents [5,15]. In clinical practice, there are also several provoking risk factors for VTE, such as hospitalization, prolonged immobility, malignancy, surgery, medical illnesses, pregnancy, and trauma [16,17], which account for up to 60% of all VTE events. However, approximately 40% of VTE cases are currently classified as unprovoked, where no trigger can be identified even despite thorough testing for thrombophilia. This highlights the importance of further searching for risk factors to better understand the occurrence of these events [17].

In this review, we discuss the potential role of accelerated biological aging in VTE, critically examine the advantages and disadvantages of the methods for investigating biological aging, in particular biomarkers of biological aging, and discuss possibilities for research in VTE.

## 2. Aging and Changes in Coagulation

Physiological hemostasis is a balance and ensures the prevention of excessive bleeding and the formation of blood clots [5,18]. This balance is maintained by complex interactions of various components, including coagulation factors, the fibrinolytic system, platelets, and the vessel wall [5,18,19]. Normally, natural inhibitors regulate clot formation, therefore preventing clot expansion. However, this balance can be disrupted when there is an increase in procoagulant activity or a decrease in the level of natural inhibitors, as might happen during aging [5,18]. For instance, with increasing age, there is an elevation in plasma levels of coagulation proteins such as factor VII, factor VIII, D-dimer, and fibrinogen, as well as antifibrinolytic factors like plasminogen activator inhibitor [PAI]-1 [20,21,22,23]. However, this happens without a proportional rise in anticoagulant proteins, such as protein C, protein S, antithrombin, and tissue factor pathway inhibitors [20,21,22,23]. Further, aging is associated with an increased thrombin generation potential, and major alterations in the fibrinolytic system resulting in reduced fibrinolytic activity [24]. This shift towards a more procoagulant state may predispose aging individuals to a higher risk of thrombosis. Besides alterations in coagulation and fibrinolysis, platelet function and changes in blood vessels also lead to a higher tendency for thrombosis in the elderly. In arterial disease, it has been reported that age-related alterations are caused by the degeneration of elastic fibers and an increase in collagen, leading to the stiffening and dilation of arteries [25].

### Inflammation and Aging

Aging is associated with a chronic, low-grade inflammatory state termed “inflammaging,” which contributes to the pathogenesis of several age-related diseases, including cardiovascular conditions and thrombosis. This persistent inflammation arises from a combination of immunosenescence, cellular damage, and metabolic dysregulation. Its effects on the vascular system and coagulation pathways establish inflammation as a key player in aging-related thrombosis. For example, aging is associated with higher levels of reactive oxygen species (ROS), due to the impairment of mitochondria, which can trigger changes leading to an imbalance between clotting and bleeding. These changes may affect blood coagulation, vessel function, and blood flow regulation [5,18]. However, ROS also activates the NLRP3 inflammasome, a key driver of inflammation. ROS acts both as a trigger and modulator of the inflammasome, influencing its activation through mitochondrial dysfunction and thiol oxidation [26]. Specifically, ROS generation can destabilize mitochondrial membranes and oxidize redox-sensitive cysteine residues on NLRP3, promoting its conformational changes and assembly into the active inflammasome complex. This dual role underscores ROS’s critical function in linking oxidative stress to inflammatory responses [26].

Furthermore, inflammaging is especially characterized by elevated levels of pro-inflammatory cytokines (e.g., IL-6, TNF-α, and IL-1β) and acute-phase proteins such as C-reactive protein (CRP) [27]. In this context, fibrinogen has a crucial role, as its increase is triggered by inflammation. Studies have consistently found that especially the increase in fibrinogen levels with advanced age leads to a greater risk of cardiovascular events. The exact mechanisms underlying the increased cardiovascular risk remain unclear. However, chances in clot formation properties, blood viscosity, and activation of platelet aggregation might mechanistically be involved [28]. Fibrinogen levels rise in response to IL-6, which is closely linked to aging. The rise in IL-6 levels is a consistent change observed with aging, potentially influenced by increased catecholamines and decreased sex steroid levels in elderly individuals. Furthermore, IL-6 demonstrates prothrombotic effects such as increasing platelet counts, promoting platelet aggregation by modulating thromboxane A2 and other signaling pathways, and inducing thrombosis in animal studies [29,30,31,32,33,34]. Additionally, IL-6 induces the secretion of C-reactive protein (CRP), which further activates the complement system and induces monocytes to express tissue factor (TF), contributing to atherosclerosis [35,36,37,38]. Moreover, chronic inflammation damages the endothelium, promoting endothelial activation and the expression of prothrombotic molecules such as TF, von Willebrand factor (vWF), and P-selectin. There are several age-related diseases, where thrombosis is a major concern, directly influenced by inflammation, like coronary artery disease, VTE, cancer-associated thrombosis, and neurovascular diseases. Additionally, inflammaging often acts on comorbidities that may increase the thrombotic risk such as diabetes, obesity, and chronic kidney disease. Overall, there is a complex interplay between coagulation and inflammation, suggesting a potential feedback loop involving thrombosis and inflammatory processes [35,39].

## 3. Biological Aging and Biomarkers of Biological Aging

The process of biological aging is highly complex and influenced by the interplay of various dysregulated cellular and biochemical mechanisms [40,41]. Aging has an impact on almost all biological functions, and the measurement of several indicators has been suggested, ranging from visible signs like greying hair to molecular changes such as telomere length [42,43].

The definition of a biomarker indicates various processes within an individual, including normal functions, pathological conditions, or responses to treatments or exposures [44]. Recognition of biomarkers of aging dates back to the 1960s, when it became evident that aging is not a fixed process but rather modifiable [45]. Over the past few decades, there have been developments in molecular and omics biomarkers of aging, offering promising options [40,46,47]. However, there is currently no consensus on the methods for evaluating and validating these biomarkers, nor is there standardization in their application [47,48].

**Figure 1:**
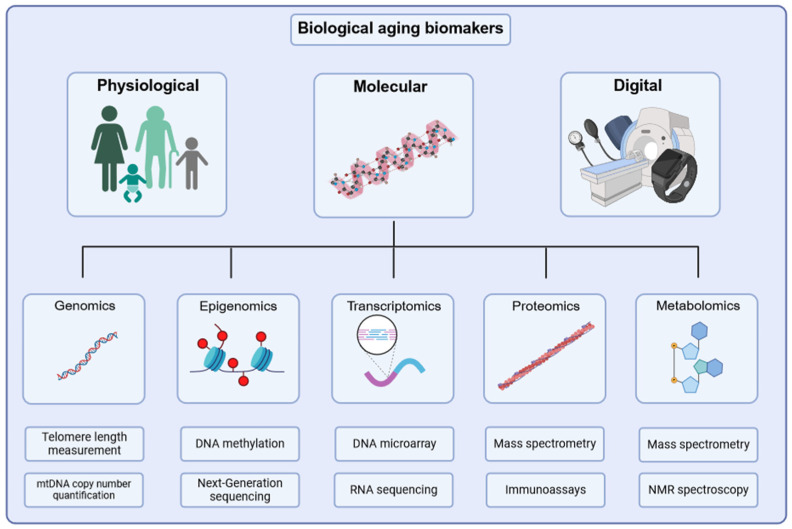
Biological aging biomarkers can be grouped into three categories: physiological, molecular, and digital biomarkers. Molecular biomarkers include genomics, epigenomics, transcriptomics, proteomics, and metabolomics studies. Figure was created with BioRender.com.

Biological aging lacks a proper definition as the term aging is used to refer to various mechanisms [49,50]. Aging involves changes over time, leading to the breakdown of multiple physiological systems. These changes occur non-uniformly across time, cell types, organ systems, individuals, and populations. Individual factors such as genetics, lifetime exposures, and disease also influence the aging process [50,51,52,53,54,55]. Biomarkers of aging are categorized into physiological, molecular, and digital biomarkers (Figure 1) [56,57]. Molecular biomarkers, the largest category, are based on omics or specific molecules, while physiological biomarkers measure functional performance or physical characteristics [56]. Compared with that, a digital biomarker refers to data generated by digital devices, such as sensors or wearables, that can reflect biological processes linked to aging. However, there is a lot of uncertainty in predicting aging progression with biomarkers, and more research is needed.

Over the past decade, advancements in omics approaches have allowed researchers to delve deep into the molecular complexity of biological aging. Techniques like high-throughput genomics, epigenomics, transcriptomics, proteomics, and metabolomics analysis enable the identification and quantification of thousands of epigenetic markers, transcripts, proteins, and metabolites, revealing the molecular changes that organisms undergo with age [58,59]. However, omics results present new challenges in the analysis and interpretation of data.

## 4. Genomics

### 4.1. Telomere Length Measurement

An approach to measuring biological aging is the measurement of telomere length. Telomeres, composed of repeating TTAGGG sequences, are protective structures at the ends of chromosomes. They prevent chromosome ends from being recognized as double-stranded DNA breaks by forming specialized T-loop structures, bound by shelterin proteins [60,61]. Telomeres undergo progressive shortening in dividing somatic cells, a process associated with aging. While telomerase, an enzyme expressed during human development, is typically inactive in most adult tissues, leading to telomere loss, even regulated telomerase expression in normal stem cells is often insufficient to prevent telomere reduction. Progressive telomere shortening can lead to replicative senescence and cell-cycle arrest in vitro and, in combination with other alterations, can result in chromosome instability and cancer development. Most cancer cells reactivate telomerase to maintain stable short telomeres and divide indefinitely [62,63,64,65,66]. Furthermore, telomere shortening and reduced telomerase activity, hallmarks of biological aging, also impact megakaryocyte differentiation and platelet function. Telomere attrition in megakaryocytes leads to replicative senescence, reducing their proliferative capacity and altering platelet production [67,68]. Aging also promotes oxidative stress and inflammation, further impairing megakaryocyte function and generating hyperactive platelets with increased aggregation potential. These dysregulated platelets play a pivotal role in thrombus formation, highlighting a direct link between aging-related telomere dynamics and thrombosis [68,69].

Understanding telomere length measurement methods is crucial because short telomeres limit cell division in somatic cells, as well as in stem cells. Additionally, genetic disorders affecting telomere shortening can lead to an earlier onset of various diseases [70,71]. Telomere length measurements can help predict the onset of certain genetic and age-related pathologies. However, lifestyle factors such as obesity, smoking, lack of exercise, and chronic stress can influence telomere length in peripheral blood leukocytes, correlating with age-associated diseases such as infertility, arthritis, diabetes, cancer, cardiovascular, and neurodegenerative diseases [71,72,73,74,75]. Various methods exist for studying telomere biology, each with advantages and disadvantages. These include quantitative polymerase chain reaction (qPCR), terminal restriction fragment analysis via Southern blot analysis, quantitative fluorescence in situ hybridization, and single-molecule real-time sequencing. Additionally, DNA methylation estimators, which assess biological aging, may indirectly relate to telomere biology but are primarily used to measure aging processes [76,77,78,79,80].

The association between telomere length measurement and VTE remains unclear. Robert Y.L. Zee could not find any correlation between relative telomere length and the risk of VTE incidents [81]. Our group also could not find differences in telomere length between patients with recurrent VTE and healthy controls [82]. Further exploration into telomere length as a biological aging biomarker for VTE is needed.

### 4.2. Mitochondrial DNA Copy Number

Another method to measure biological aging is the measurement of mitochondrial DNA copy number (mtDNA-CN). mtDNA-CN serves as a biomarker of mitochondrial function, correlating with overall mortality and various age-related diseases like cardiovascular diseases and cancer [83]. Mitochondria play an important role in energy production, bioenergetic regulation, and cell signaling, making them essential to cellular health [84]. Mitochondrial dysfunction, characterized by ineffective oxidative phosphorylation and increased ROS, contributes to aging and is linked to chronic diseases [85,86]. mtDNA-CN reflects energy reserves, oxidative stress, and mitochondrial membrane potential [87]. There are certain genetic factors and environmental exposures that influence mtDNA-CN [88]. In in vitro and cancer tissues, the reduction in mtDNA-CN correlates with altered mitochondrial function, affecting cellular energy production and inflammation. Mitochondrial dysfunction in whole blood may modulate immune responses, affecting macrophage polarization and chronic inflammatory processes in cardiovascular, kidney, and liver diseases [89,90,91].

The measurement of mtDNA-CN can be performed with isolated DNA from peripheral blood and other tissues [92,93]. Reduction in mtDNA-CN correlates with decreased expression of vital mitochondrial proteins, altered cellular morphology, and impaired respiratory enzyme activity [94]. Reduced levels have also been linked to increased oxidative stress, contributing to various diseases including CVD, liver disease, aging-related diseases, and neurodegeneration. In CVD, decreased mtDNA-CN is associated with the activation of proatherogenic genes, atherogenesis, plaque instability, and low-density lipoprotein (LDL) oxidation [95,96,97,98,99,100,101,102]. Despite its clinical potential, reliable quantification of mitochondrial dysfunction remains lacking. qPCR has been the gold standard for mtDNA-CN measurement, comparing mitochondrial to nuclear gene copies for relative quantification [92]. Quantifying mtDNA-CN in clinical practice is challenging due to technical limitations, as well as biological factors, like age- or disease-related changes and tissue-specific variability. Therefore, standardization by sex, age, platelet count, and white blood cell count is necessary due to these tissue-specific variations and age-related declines [103,104]. The complexity of mtDNA-CN quantification makes it difficult to ensure accuracy and high costs limit routine accessibility.

However, large-scale studies have shown that lower mtDNA-CN is inversely associated with prevalent and incident CVD, coronary heart disease (CHD), stroke, and sudden cardiac death (SCD), independent of traditional risk factors [93,105]. In the case of VTE, Nymberg et al. aimed to investigate the potential association between mtDNA-CN and incident VTE in middle-aged women [106]. The study encompassed 6917 women aged 50–64 years, tracked over a span of 20 years within the Women’s Health in the Lund Area (WHILA) study. However, no association of mtDNA-CN with VTE risk was observed. In contrast, our group could demonstrate that patients with recurrent VTE had significantly lower mtDNA levels compared to healthy controls [82]. The adjustment for age, sex, BMI, and smoking revealed that mtDNA copy number was independently associated with VTE risk. We also observed that mtDNA-CN differed between women and men; women had higher levels of mtDNA-CN than men did. These results indicate an association of biological aging with the risk of VTE [82].

### 4.3. Cell Senescence

Another context of biological aging noteworthy to mention is cell senescence, as VTE is increasingly recognized as both a consequence and contributor to cellular senescence. Senescence is known as cell-cycle arrest in the G1 or possibly G2 phase, preventing the proliferation of damaged cells [107,108]. On the one side, senescence acts, combined with apoptosis, during normal development to facilitate embryonic morphogenesis. In adult tissues, the senescence response is triggered by various forms of damage, acting to suppress transformed, dysfunctional, or aged cells. However, with aging, senescent cells gradually accumulate [109,110]. Interestingly, one reason causing senescence is the damage of telomeres. For example, cells that lack shelterin components suffer DNA damage, which results in senescence induction.

It is known that pro-inflammatory and hypercoagulable states become more prevalent with age, not only promoting thrombotic events but also accelerating cellular aging. In their study, Nguyen et al. identified that the tissue factor pathway plays a crucial role in linking inflammation and thrombosis to cellular senescence [111]. Specifically, the tissue factor (TF) itself, a glycoprotein present in the vascular wall under physiological conditions, becomes upregulated in response to inflammatory signals. This upregulation is often mediated by cytokines such as IL-1β and TNF-α, which then activates hemostasis, leading to thrombin generation. Thrombin, in turn, activates protease-activated receptors (PARs) on endothelial cells, which can advance endothelial cell senescence by increasing reactive oxygen species (ROS) and nitric oxide (NO) imbalance, thus, potentially elevating the risk for VTE [112]. Poredos and Jezovnik observed that factors commonly associated with aging, such as chronic inflammation, significantly contribute to endothelial damage, which, in turn, accelerates cellular senescence and increases the risk of thrombotic events [113]. Elevated levels of fibrinogen and other coagulation factors in older populations promote platelet aggregation and adhesion to the vascular endothelium, fostering conditions favorable for VTE and perpetuating endothelial inflammation and dysfunction. These age-related vascular changes create a cycle that embeds senescence within vascular cells and heightens thrombotic risk [114]. Beyond inflammation, physical factors such as changes in shear stress, oxidative stress, and the accumulation of senescent immune cells also contribute to a procoagulant environment, which, in turn, accelerates cellular aging. Konieczyńska et al. emphasized that reduced shear stress and increased oxidative stress in the aging vasculature led to the upregulation of von Willebrand factor (vWF) and other procoagulant molecules [115]. This environment promotes hypercoagulability and, ultimately, endothelial cell senescence, increasing VTE risk [115]. Synoptically, VTE risk in aging populations is not only a result of physical stasis but may also be influenced by the biological aging processes of vascular and immune cells, with pro-inflammatory and prothrombotic states promoting cellular senescence.

## 5. Epigenomics

### 5.1. DNA Methylation

The accumulation of epigenetic modifications during aging contributes to various age-related diseases. Research has shown common changes in DNA methylation patterns throughout the genome in response to aging across different species [41,116]. These age-associated epigenetic modifications can occur throughout the body or be restricted to specific tissues or cell types. Additionally, age-related alterations in DNA methylation are observed in germ cells and may potentially be passed down to offspring [117,118]. The nature of DNA lies in the dynamic interplay between the genetic sequence and the epigenome. Environmental factors influence gene expression through mechanisms such as DNA methylation, hydroxymethylation, histone modifications, alternative splicing, and others [119].

DNA methylation is a well-known covalent epigenetic modification, meaning the addition of a methyl group to the C-5 position of cytosine by DNA methyltransferase. Both methylation and demethylation processes are crucial, not only for regulating transcription but also for developmental processes and cell differentiation [118,119,120]. DNA methylation influences various biological processes, including gene regulation, genomic imprinting, cancer, and X chromosome inactivation [121,122,123,124,125,126]. While a significant amount of cytosine–phosphate–guanine (CpG) sites in mammalian cells are typically methylated, DNA methylation levels decrease with age, leading to loss of transcriptional control and contributing to age-related effects [127]. These age-related changes in DNA methylation can be utilized to predict the age in humans [128,129]. Recent studies have shown that DNA methylation measurements can serve as effective tools for predicting age, sometimes outperforming telomere length-based models in accuracy [129]. DNA methylation-based age prediction models can not only estimate chronological age but also provide insights into biological aging [130,131]. The onset of Next-generation Sequencing (NGS) has enabled a methylation-wide association study (MWAS) with high resolution at both single-base-pair and single-cell levels [132]. MWAS has become a standard tool for identifying new genes and methylation markers potentially linked to environmental and genetic risk factors [133]. Research on methylation markers in peripheral blood DNA is an innovative approach to investigating the etiology of complex diseases [132].

Unfortunately, the role of DNA methylation in venous thromboembolism (VTE) remains unclear. However, aberrant DNA methylation patterns can influence the expression of genes associated with coagulation and vascular function. For example, the overexpression of TF, a key initiator of coagulation, may result from methylation changes in the regulatory region [134,135]. Additionally, the elevation of PAI-1 caused by hypermethylation of repressors leads to impaired fibrinolysis and clot persistence [136]. Moreover, the hypermethylation of NO genes may reduce their expression, impairing endothelial function and promoting thrombosis. The methylation changes of anti-inflammatory cytokines and pro-inflammatory mediators, like IL-6, further support a prothrombotic environment [137]. Nevertheless, the exploration into the potential of DNA methylation as a diagnostic or preventive biomarker for VTE is needed. However, studies have indicated that the expression levels of certain genes encoding hemostatic proteins, such as FVII, FVIII, and tissue-type plasminogen activator, can be influenced by DNA methylation mechanisms, leading to alterations in their plasma concentrations under various conditions [138,139,140]. One pioneering study conducted MWAS using whole blood cells to explore potential correlations between methylation markers and quantitative characteristics related to the coagulation cascade [141]. Another study found that DNA methylation did not influence the FV Leiden variant and did not provide an explanation for its incomplete penetrance [138].

### 5.2. Transcriptomics, Proteomics, and Metabolomics

Methods in transcriptomics, proteomics, and metabolomics are also promising to investigate biological aging. In the case of transcriptomics, RNA gene expression levels were used to construct aging clocks, offering a more direct link between aging and genes. In 2015, Peters et al. developed a transcriptomic clock, using gene expression data from peripheral blood mononuclear cells across multiple large cohorts [142]. However, the accuracy of this transcriptomic clock in predicting chronological age varied across the cohorts [142]. These variations may have resulted from the integration of microarray and sequencing data from diverse platforms, leading to technical noise in the dataset.

The field of proteomics has made significant progress during the past decade. Mass spectrometry-based, antibody-based, and aptamer-based proteomics helped with the quantification of thousands of proteins in a single sample [143,144]. Several studies using different proteomic technologies revealed age-related changes in thousands of proteins in human plasma and cerebrospinal fluid. These findings led to the development of multiple proteomic aging clocks [145,146,147]. Previous studies utilized standard approaches to design proteomic clocks and observed associations with physiological and clinical aging features [148,149,150]. Studies have also revealed that several proteins identified in plasma proteomic aging clocks directly influence lifespan, while hundreds are biologically linked to the health status of various organs [151,152]. The possibility of associating the data with organ function enables the exploration of how aging manifests differently across tissues and cell types. Proteomics is an interesting approach for investigating biological aging in VTE, as the application reveals a lot of information. Additionally, blood plasma contains proteins from all organs and cell types, which is an advantage, as blood is easy to obtain [41,151].

Another omics approach that is involved in the field of biological aging is metabolomics. The usage of mass spectrometry and nuclear magnetic resonance (NMR) methods has the capability to identify hundreds to thousands of metabolites in human plasma, aiming to unravel their roles in aging [153,154]. An interesting NMR biobank study developed a metabolomic clock based on 56 measurable plasma metabolites. This clock was then used to investigate relationships between metabolomic age, cardiovascular phenotypes, and mortality [155]. Accelerated metabolomic age was linked with cardiovascular risk factors, cardiovascular disease risk, and all-cause mortality risk in independent prospective cohorts. Despite challenges, such as data reliability and variability, the strong connections between metabolism and aging provide a good base for the further exploration and development of metabolomic clocks [156,157]. Like most other omics approaches, metabolomics has not received much attention in connection with VTE and biological aging.

Although transcriptomics, proteomics, and metabolomics have been used in the field of biological aging, they have not been widely applied in studies on VTE. These methods hold promise in identifying novel biomarkers associated with VTE (Table 1).

The current findings in various omics fields have the potential to reveal differences between VTE patients and healthy individuals and identify novel biomarkers for VTE, which could give further insights into the molecular mechanisms of VTE. Furthermore, investigating VTE patients at different stages (acute, post-acute, and chronic phases of VTE), can contribute to understanding the disease biology and monitor recovery from VTE on a molecular level, with a focus on biological aging.

The broad overview of omics approaches allows a better understanding of complex biological processes. Omics technologies enable high-throughput analysis, allowing the simultaneous examination of thousands to millions of data points in a single experiment [46]. This possibility accelerates research and helps in the discovery of biomarkers, therapeutic targets, and disease mechanisms. The application of omics data with clinical information and environmental factors generates multidimensional datasets, enhancing our understanding of disease etiology, progression, and treatment response [46]. This translational potential of omics findings can be applied from bench to bedside. By bridging basic research with clinical practice, omics studies contribute to the development of new diagnostics, therapeutics, and interventions, with the goal to improve patient outcomes [46]. In the context of VTE, combining risk factor research with biological aging studies holds promise for future research. Valuable insights into disease mechanisms can be gained and, ultimately, personalized treatment approaches could be developed.

## 6. Future Directions

Aging is a well-established risk factor for venous thromboembolism (VTE), encompassing both deep vein thrombosis and pulmonary embolism. The increased risk associated with aging arises from physiological changes, such as increased procoagulant activity, endothelial dysfunction, and chronic inflammation. While clinical factors such as immobility, comorbidities, and prior VTE events are commonly used for stratification, biomarkers provide an opportunity for a more precise and individualized risk assessment. There are already existing biomarkers that improve risk prediction and reflect a prothrombotic state. For example, the incorporation of molecular indicators of hypercoagulability, endothelial dysfunction, or inflammation like D-dimer, which is a degradation product of fibrin, and is widely recognized for its role in ruling out acute VTE. Elevated baseline levels in older individuals could serve as a stratifying factor for future risk. Aging is also linked to an increase in prothrombotic markers such as thrombin–antithrombin complexes, factor VIII, and fibrinogen. Tracking these markers may help identify individuals who are predisposed to thrombosis but have not yet exhibited clinical symptoms, allowing for preventive interventions. Furthermore, inflammation also plays a significant role in VTE risk among the elderly. Biomarkers like CRP and IL-6 can provide insights into the inflammatory milieu, which interacts with coagulation pathways to promote thrombosis. Furthermore, the inclusion of potential biomarkers of biological aging in VTE risk stratification models may enhance predictive accuracy and personalize prevention strategies.

- Telomere length: Shorter telomere length correlates with increased cardiovascular and thrombotic risks. As telomere length shortens, measuring telomere length could stratify aging individuals into higher or lower thrombotic risk categories, particularly when combined with other clinical factors.
- mtDNA copy number: Oxidative stress from dysfunctional mitochondria increases endothelial damage and platelet activation, promoting thrombosis. Monitoring mtDNA copy numbers could help identify aging individuals with mitochondrial dysfunction and a heightened tendency for thromboembolic events.
- Cellular senescence: Cellular senescence is implicated in endothelial dysfunction and vascular stiffening, both of which increase thrombosis risk. Quantifying markers of senescence could help stratify VTE risk based on the biological burden of senescent cells.
- DNA methylation: Specific methylation changes may influence genes related to coagulation, inflammation, and endothelial function. For instance, hypermethylation of anti-inflammatory pathways or hypomethylation of pro-inflammatory genes can predispose to thrombosis. Integrating epigenetic clocks into risk models could identify biologically older individuals at higher risk of VTE, independent of chronological age.
- Transcriptomics: Differential expression of genes involved in coagulation (e.g., fibrinogen, thrombin receptors) or inflammation (e.g., IL-6, TNF-α) could signify increased VTE risk. Transcriptomic signatures might refine VTE risk models by detecting subclinical prothrombotic states in aging individuals.
- Proteomics: Age-related shifts in protein profiles, including increased levels of the fibrinogen, factor VIII, and von Willebrand factor, are linked to hypercoagulability. Comprehensive proteomic profiling could help identify individuals with an elevated protein signature indicative of VTE risk.
- Metabolomics: Alterations in lipid metabolism, amino acid pathways, and oxidative stress metabolites are associated with VTE. Aging individuals often show dysregulated metabolic profiles, which may contribute to thrombotic risk. The analysis of metabolomic signatures can capture the systemic metabolic environment, providing a VTE risk in the context of aging.

Integrating these biological aging biomarkers into VTE risk stratification could revolutionize preventive strategies and allow for earlier and more targeted prophylaxis. As the use of all these novel methods requires specific knowledge and bioinformatic expertise, interdisciplinary collaborations are essential to investigate biological aging and the mechanisms underlying biological aging in VTE.

## Figures and Tables

**Table 1:** Omics approaches in VTE research.

Examples of Transcriptomic-Based Thrombosis Research	
Lindström et al., 2019 [158]	Besides the genome-wide association study (GWAS), Lindström et al. integrated a transcriptome-wide association study (TWAS) of VTE [158]. In this comprehensive study, the authors identified 34 distinct genetic signals associated with VTE risk, with 14 being newly discovered. This included 11 newly associated genetic loci and 3 new independent signals within known genes. Additionally, TWAS analysis revealed 5 more genetic loci related to VTE predisposition. Some of these associations were found to be influenced by nearby gene expression. This study also introduced a Genetic Risk Score based on 37 VTE-susceptibility variants, identifying individuals at high risk. Mendelian randomization analyses suggested that certain blood characteristics may increase the risk of VTE [158].
Thibord et al., 2023 [159]	Thibord et al. performed a cross-ancestry GWAS meta-analysis involving over 55,000 participants with VTE of diverse ancestries and identified a total of 135 independent genomic loci significantly associated with VTE risk [159]. This included 48 novel associations, with 34 replicating after stringent testing corrections. Through TWAS, they also discovered 31 novel transcript associations and 8 new candidate genes with protein quantitative trait locus Mendelian randomization analyses [159].
**Examples of Proteomics-Based Thrombosis Research**	
Bruzelius et al., 2016 [160]	This study aimed to discover plasma biomarkers for predicting VTE risk [160]. Using affinity reagents and multiplexed immunoassays, plasma samples from two studies were analyzed. Significant associations were found between VTE and plasma levels of Human Immunodeficiency Virus Type I Enhancer Binding Protein 1 (HIVEP1), von Willebrand Factor (VWF), Glutathione Peroxidase 3 (GPX3), and Platelet-derived Growth Factor Subunit B (PDGFB). Replication in another study confirmed associations of VWF and PDGFB with VTE. PDGFΒ’s association was validated by further analyses. Bruzelius et al. demonstrated the potential of high-throughput plasma proteomic profiling to identify VTE biomarkers, highlighting a novel association of PDGFB with VTE [160].
Yuan et al., 2024 [161]	Yuan et al. performed a network Mendelian randomization (MR) analysis [161]. They showed associations between metabolic factors, proteins, and VTE using GWAS data from 35,559 individuals. Yuan et al. could identify over 30 proteins that may contribute to VTE development. To analyze the contribution of protein pathways with 2-stage MR network analysis to link modifiable factors, like obesity, smoking, and insomnia, to VTE, they could show that there are two key mediators, low-density lipoprotein receptor-related protein 12 (LRP-12) and coagulation factor XI (FXI) [161].
Ten Cate et al., 2021 [162]	This study investigated molecular differences between isolated PE and PE associated with DVT, as well as isolated DVT [162]. Using targeted proteomics, shared and distinct protein signatures were identified for each phenotype. Shared processes included inflammation and oxidative stress response, while isolated PE exhibited a unique protein signature involving five specific proteins (interferon-γ, glial-cell-line-derived neurotrophic growth factor, polypeptide N-acetyl-galactosaminyl- transferase 3, peptidyl arginine deiminase type-2, and interleukin-15 receptor subunit α. These findings were externally validated and were predictive of incident of isolated PE. Overall, this study suggests an involvement of noncanonical pathways in isolated PE pathophysiology [162].
**Examples of Metabolomics-Based Thrombosis Research**	
Jiang et al., 2021 [163]	In this study, 211 metabolites were measured for each participant, alongside detailed lifestyle data [163]. Logistic regression and enrichment analyses were conducted to identify metabolites and biological categories associated with incident VTE risk, while considering key confounders like age, sex, smoking, alcohol intake, BMI, and comorbid conditions. In the first step, 60 metabolites were associated with VTE or pulmonary embolism, with enrichment in the metabolomics category diacylglycerols (DAGs). However, after adjusting for multiple testing only C5 carnitine remained significantly associated with VTE. And after further adjustment for BMI, no significant associations between metabolites and VTE remained [163].
Febra et al., 2024 [164]	In this observational trial, 62 patients presenting with suspected acute deep vein thrombosis (DVT) or pulmonary embolism (PE) were evaluated [164]. Among them, 50 were diagnosed with acute VTE, while 12 had non-acute VTE conditions. Metabolomics analysis of plasma and red blood cells (RBCs) identified distinct metabolic signatures associated with acute VTE. Plasma analysis revealed 23 significantly different molecules, with the d-glutamine and d-glutamate pathway having the strongest association. RBCs displayed a specific metabolomic signature in VTE patients, primarily characterized by alterations in purine metabolism. Three metabolites, adenosine 3′,5′-diphosphate, glutathione, and adenine, showed high performance in distinguishing patients with acute and non-acute VTE [164].

## Data Availability

No new data were created or analyzed in this study. Data sharing is not applicable to this article.

## References

[1] Wang Q., Zennadi R. (2020). Oxidative Stress and Thrombosis during Aging: The Roles of Oxidative Stress in RBCs in Venous Thrombosis. Int. J. Mol. Sci..

[2] Gregson J., Kaptoge S., Bolton T., Pennells L., Willeit P., Burgess S., Bell S., Sweeting M., Rimm E.B., Kabrhel C. (2019). Cardiovascular Risk Factors Associated With Venous Thromboembolism. JAMA Cardiol..

[3] Goldhaber S.Z., Bounameaux H. (2012). Pulmonary embolism and deep vein thrombosis. Lancet.

[4] Cohen A.T., Agnelli G., Anderson F.A., Arcelus J., Bergqvist D., Brecht J.G., Greer I.A., Heit J.A., Hutchinson J.L., Kakkar A.K. (2007). Venous thromboembolism (VTE) in Europe. The number of VTE events and associated morbidity and mortality. Thromb. Haemost..

[5] Favaloro E.J., Franchini M., Lippi G. (2014). Aging hemostasis: Changes to laboratory markers of hemostasis as we age—A narrative review. Semin. Thromb. Hemost..

[6] Abbate R., Prisco D., Rostagno C., Boddi M., Gensini G.F. (1993). Age-related changes in the hemostatic system. Int. J. Clin. Lab. Res..

[7] Hamczyk M.R., Nevado R.M., Barettino A., Fuster V., Andrés V. (2020). Biological Versus Chronological Aging: JACC Focus Seminar. J. Am. Coll. Cardiol..

[8] Karasik D., Demissie S., Cupples L.A., Kiel D.P. (2005). Disentangling the Genetic Determinants of Human Aging: Biological Age as an Alternative to the Use of Survival Measures. J. Gerontol. Ser. A.

[9] Bafei S.E.C., Shen C. (2023). Biomarkers selection and mathematical modeling in biological age estimation. NPJ Aging.

[10] Levine M.E., Lu A.T., Quach A., Chen B.H., Assimes T.L., Bandinelli S., Hou L., Baccarelli A.A., Stewart J.D., Li Y. (2018). An epigenetic biomarker of aging for lifespan and healthspan. Aging.

[11] Kang Y.G., Suh E., Lee J.-W., Kim D.W., Cho K.H., Bae C.-Y. (2018). Biological age as a health index for mortality and major age-related disease incidence in Koreans: National health Insurance service–health screening 11-year follow-up study. Clin. Interv. Aging.

[12] Levine M.E. (2013). Modeling the rate of senescence: Can estimated biological age predict mortality more accurately than chronological age?. J. Gerontol. Ser. A Biomed. Sci. Med. Sci..

[13] Guerville F., Barreto P.D.S., Ader I., Andrieu S., Casteilla L., Dray C., Fazilleau N., Guyonnet S., Langin D., Liblau R. (2020). Revisiting the Hallmarks of Aging to Identify Markers of Biological Age. J. Prev. Alzheimer’s Dis..

[14] Akrivou D., Perlepe G., Kirgou P., Gourgoulianis K.I., Malli F. (2022). Pathophysiological Aspects of Aging in Venous Thromboembolism: An Update. Medicina.

[15] Dolcini J., Wu H., Nwanaji-Enwerem J.C., Kiomourtozlogu M.-A., Cayir A., Sanchez-Guerra M., Vokonas P., Schwarz J., Baccarelli A.A. (2020). Mitochondria and aging in older individuals: An analysis of DNA methylation age metrics, leukocyte telomere length, and mitochondrial DNA copy number in the VA normative aging study. Aging.

[16] Vaiserman A., Krasnienkov D. (2021). Telomere Length as a Marker of Biological Age: State-of-the-Art, Open Issues, and Future Perspectives. Front. Genet..

[17] Kane A.E., Sinclair D.A. (2019). Epigenetic changes during aging and their reprogramming potential. Crit. Rev. Biochem. Mol. Biol..

[18] Versteeg H.H., Heemskerk J.W.M., Levi M., Reitsma P.H. (2013). New Fundamentals in Hemostasis. Physiol. Rev..

[19] Bochenek M.L., Schütz E., Schäfer K. (2016). Endothelial cell senescence and thrombosis: Ageing clots. Thromb. Res..

[20] Hager K., Setzer J., Vogl T., Voit J., Platt D. (1989). Blood coagulation factors in the elderly. Arch. Gerontol. Geriatr..

[21] Tofler G.H., Massaro J., Levy D., Mittleman M., Sutherland P., Lipinska I., Muller J.E., D’agostino R.B. (2005). Relation of the Prothrombotic State to Increasing Age (from the Framingham Offspring Study). Am. J. Cardiol..

[22] Bauer K.A., Weiss L.M., Sparrow D., Vokonas P.S., Rosenberg R.D. (1987). Aging-associated changes in indices of thrombin generation and protein C activation in humans. Normative Aging Study. J. Clin. Investig..

[23] Lowe G.D.O., Lee A.J., Rumley A., Price J.F., Fowkes F.G.R. (1997). Blood viscosity and risk of cardiovascular events: The Edinburgh Artery Study. Br. J. Haematol..

[24] Mari D., Ogliari G., Castaldi D., Vitale G., Bollini E.M., Lio D. (2008). Hemostasis and ageing. Immun. Ageing.

[25] Lakatta E.G., Mitchell J.H., Pomerance A., Rowe G.G. (1987). Human aging: Changes in structure and function. J. Am. Coll. Cardiol..

[26] Abais J.M., Xia M., Zhang Y., Boini K.M., Li P.-L. (2015). Redox regulation of NLRP3 inflammasomes: ROS as trigger or effector?. Antioxid. Redox Signal.

[27] Rea I.M., Gibson D.S., McGilligan V., McNerlan S.E., Alexander H.D., Ross O.A. (2018). Age and Age-Related Diseases: Role of Inflammation Triggers and Cytokines. Front. Immunol..

[28] Tracy R., Bovill E. (1992). Thrombosis and cardiovascular risk in the elderly. Arch. Pathol. Lab. Med..

[29] Tracy R.P. (2002). Hemostatic and Inflammatory Markers as Risk Factors for Coronary Disease in the Elderly. Am. J. Geriatr. Cardiol..

[30] Ershler W.B. (1993). Interleukin-6: A Cytokine for Gerontolgists. J. Am. Geriatr. Soc..

[31] Ershler W.B., Sun W.H., Binkley N., Gravenstein S., Volk M.J., Kamoske G., Klopp R.G., Roecker E.B., A Daynes R., Weindruch R. (1993). Interleukin-6 and aging: Blood levels and mononuclear cell production increase with advancing age and in vitro production is modifiable by dietary restriction. Lymphokine Cytokine Res..

[32] Soslau G., Morgan D.A., Jaffe J.S., Brodsky I., Wang Y. (1997). Cytokine mRNA expression in human platelets and a megakaryocytic cell line and cytokine modulation of platelet function. Cytokine.

[33] Burstein S.A. (1994). Effects of interleukin 6 on megakaryocytes and on canine platelet function. Stem Cells.

[34] Mestries J.C., Kruithof E.K., Gascon M.P., Herodin F., Agay D., Ythier A. (1994). In vivo modulation of coagulation and fibrinolysis by recombinant glycosylated human interleukin-6 in baboons. Eur. Cytokine Netw..

[35] Ritchie D.G., Levy B.A., A Adams M., Fuller G.M. (1982). Regulation of fibrinogen synthesis by plasmin-derived fragments of fibrinogen and fibrin: An indirect feedback pathway. Proc. Natl. Acad. Sci. USA.

[36] Pepys M.B., Baltz M.L. (1983). Acute phase proteins with special reference to C-reactive protein and related proteins (pentaxins) and serum amyloid A protein. Adv. Immunol..

[37] Cermak J., Key N.S., Bach R.R., Balla J., Jacob H.S., Vercellotti G.M. (1993). C-reactive protein induces human peripheral blood monocytes to synthesize tissue factor. Blood.

[38] Bataille R., Klein B. (1992). C-reactive protein levels as a direct indicator of interleukin-6 levels in humans in vivo. Arthritis Rheum..

[39] Wang Y., Fuller G.M. (1991). The putative role of fibrin fragments in the biosynthesis of fibrinogen by hepatoma cells. Biochem. Biophys. Res. Commun..

[40] Kennedy B.K., Berger S.L., Brunet A., Campisi J., Cuervo A.M., Epel E.S., Franceschi C., Lithgow G.J., Morimoto R.I., Pessin J.E. (2014). Geroscience: Linking aging to chronic disease. Cell.

[41] López-Otín C., Blasco M.A., Partridge L., Serrano M., Kroemer G. (2013). The hallmarks of aging. Cell.

[42] Bobrov E., Georgievskaya A., Kiselev K., Sevastopolsky A., Zhavoronkov A., Gurov S., Rudakov K., Tobar M.d.P.B., Jaspers S., Clemann S. (2018). PhotoAgeClock: Deep learning algorithms for development of non-invasive visual biomarkers of aging. Aging.

[43] Sanders J.L., Newman A.B. (2013). Telomere length in epidemiology: A biomarker of aging, age-related disease, both, or neither?. Epidemiol. Rev..

[44] Califf R.M. (2018). Biomarker definitions and their applications. Exp. Biol. Med..

[45] Comfort A. (1969). Test-battery to measure ageing-rate in man. Lancet.

[46] Rutledge J., Oh H., Wyss-Coray T. (2022). Measuring biological age using omics data. Nat. Rev. Genet..

[47] Partridge L., Fuentealba M., Kennedy B.K. (2020). The quest to slow ageing through drug discovery. Nat. Rev. Drug Discov..

[48] Lara J., Cooper R., Nissan J., Ginty A.T., Khaw K.-T., Deary I.J., Lord J.M., Kuh D., Mathers J.C. (2015). A proposed panel of biomarkers of healthy ageing. BMC Med..

[49] Cohen A.A., Kennedy B.K., Anglas U., Bronikowski A.M., Deelen J., Dufour F., Ferbeyre G., Ferrucci L., Franceschi C., Frasca D. (2020). Lack of consensus on an aging biology paradigm? A global survey reveals an agreement to disagree, and the need for an interdisciplinary framework. Mech. Ageing Dev..

[50] Galkin F., Mamoshina P., Aliper A., de Magalhães J.P., Gladyshev V.N., Zhavoronkov A. (2020). Biohorology and biomarkers of aging: Current state-of-the-art, challenges and opportunities. Ageing Res. Rev..

[51] Gladyshev V.N. (2016). Aging: Progressive decline in fitness due to the rising deleteriome adjusted by genetic, environmental, and stochastic processes. Aging Cell.

[52] Nie C., Li Y., Li R., Yan Y., Zhang D., Li T., Li Z., Sun Y., Zhen H., Ding J. (2022). Distinct biological ages of organs and systems identified from a multi-omics study. Cell Rep..

[53] Buckley M.T., Sun E.D., George B.M., Liu L., Schaum N., Xu L., Reyes J.M., Goodell M.A., Weissman I.L., Wyss-Coray T. (2023). Cell-type-specific aging clocks to quantify aging and rejuvenation in neurogenic regions of the brain. Nat. Aging.

[54] Ahadi S., Zhou W., Rose S.M.S.-F., Sailani M.R., Contrepois K., Avina M., Ashland M., Brunet A., Snyder M. (2020). Personal aging markers and ageotypes revealed by deep longitudinal profiling. Nat. Med..

[55] Cohen A.A., Legault V., Fuellen G., Fülöp T., Fried L.P., Ferrucci L. (2018). The risks of biomarker-based epidemiology: Associations of circulating calcium levels with age, mortality, and frailty vary substantially across populations. Exp. Gerontol..

[56] Xia X., Chen W., McDermott J., Han J.-D.J. (2017). Molecular and phenotypic biomarkers of aging. F1000Research.

[57] Hartmann A., Hartmann C., Secci R., Hermann A., Fuellen G., Walter M. (2021). Ranking Biomarkers of Aging by Citation Profiling and Effort Scoring. Front. Genet..

[58] Schaum N., Lehallier B., Hahn O., Pálovics R., Hosseinzadeh S., Lee S.E., Sit R., Lee D.P., Losada P.M., Zardeneta M.E. (2020). Ageing hallmarks exhibit organ-specific temporal signatures. Nature.

[59] (2020). A single-cell transcriptomic atlas characterizes ageing tissues in the mouse. Nature.

[60] Palm W., de Lange T. (2008). How shelterin protects mammalian telomeres. Annu. Rev. Genet..

[61] de Lange T. (2002). Protection of mammalian telomeres. Oncogene.

[62] Fumagalli M., Rossiello F., Clerici M., Barozzi S., Cittaro D., Kaplunov J.M., Bucci G., Dobreva M., Matti V., Beausejour C.M. (2012). Telomeric DNA damage is irreparable and causes persistent DNA-damage-response activation. Nat. Cell Biol..

[63] Zou Y., Sfeir A., Gryaznov S.M., Shay J.W., Wright W.E. (2004). Does a sentinel or a subset of short telomeres determine replicative senescence?. Mol. Biol. Cell.

[64] Hemann M.T., Strong M.A., Hao L.-Y., Greider C.W. (2001). The shortest telomere, not average telomere length, is critical for cell viability and chromosome stability. Cell.

[65] Herbig U., Jobling W.A., Chen B.P., Chen D.J., Sedivy J.M. (2004). Telomere shortening triggers senescence of human cells through a pathway involving ATM, p53, and p21(CIP1), but not p16(INK4a). Mol. Cell.

[66] von Zglinicki T., Saretzki G., Ladhoff J., di Fagagna F.D., Jackson S. (2005). Human cell senescence as a DNA damage response. Mech. Ageing Dev..

[67] Liu H., Liu J., Wang L., Zhu F. (2021). In vitro Generation of Megakaryocytes and Platelets. Front. Cell Dev. Biol..

[68] Faria A.V.S., Andrade S.S., Peppelenbosch M.P., Ferreira-Halder C.V., Fuhler G.M. (2020). Platelets in aging and cancer—“double-edged sword”. Cancer Metastasis Rev..

[69] Rojas-Sanchez G., Davizon-Castillo P., Harris J.R., Korolchuk V.I. (2023). An Insight into Platelets at Older Age: Cellular and Clinical Perspectives. Biochemistry and Cell Biology of Ageing: Part III Biomedical Science.

[70] Holohan B., Wright W.E., Shay J.W. (2014). Cell biology of disease: Telomeropathies: An emerging spectrum disorder. J. Cell Biol..

[71] Opresko P.L., Shay J.W. (2017). Telomere-associated aging disorders. Ageing Res. Rev..

[72] Shay J.W. (2016). Role of Telomeres and Telomerase in Aging and Cancer. Cancer Discov..

[73] Sampson M.J., Hughes D.A. (2006). Chromosomal telomere attrition as a mechanism for the increased risk of epithelial cancers and senescent phenotypes in type 2 diabetes. Diabetologia.

[74] Samani N.J., Boultby R., Butler R., Thompson J.R., Goodall A.H. (2001). Telomere shortening in atherosclerosis. Lancet.

[75] Fitzpatrick A.L., Kronmal R.A., Gardner J.P., Psaty B.M., Jenny N.S., Tracy R.P., Walston J., Kimura M., Aviv A. (2007). Leukocyte telomere length and cardiovascular disease in the cardiovascular health study. Am. J. Epidemiol..

[76] Cawthon R.M. (2002). Telomere measurement by quantitative PCR. Nucleic Acids Res..

[77] Cawthon R.M. (2009). Telomere length measurement by a novel monochrome multiplex quantitative PCR method. Nucleic Acids Res..

[78] Lansdorp P.M., Verwoerd N.P., van de Rijke F.M., Dragowska V., Little M.T., Dirks R.W., Raap A.K., Tanke H.J. (1996). Heterogeneity in telomere length of human chromosomes. Hum. Mol. Genet..

[79] Kimura M., Stone R.C., Hunt S.C., Skurnick J., Lu X., Cao X., Harley C.B., Aviv A. (2010). Measurement of telomere length by the Southern blot analysis of terminal restriction fragment lengths. Nat. Protoc..

[80] Canela A., Vera E., Klatt P., Blasco M.A. (2007). High-throughput telomere length quantification by FISH and its application to human population studies. Proc. Natl. Acad. Sci. USA.

[81] Zee R.Y.L., Michaud S.E., Ridker P.M. (2009). Mean telomere length and risk of incident venous thromboembolism: A prospective, nested case-control approach. Clin. Chim. Acta Int. J. Clin. Chem..

[82] Vostatek R., Hohensinner P., Nopp S., Haider P., Englisch C., Pointner J., Pabinger I., Ay C. (2023). Association of telomere length and mitochondrial DNA copy number, two biomarkers of biological aging, with the risk of venous thromboembolism. Thromb. Res..

[83] Castellani C.A., Longchamps R.J., Sun J., Guallar E., Arking D.E. (2020). Thinking outside the nucleus: Mitochondrial DNA copy number in health and disease. Mitochondrion.

[84] Wallace D.C. (1992). Diseases of the Mitochondrial DNA. Annu. Rev. Biochem..

[85] Guyatt A.L., Burrows K., Guthrie P.A., Ring S., McArdle W., Day I.N., Ascione R., Lawlor D.A., Gaunt T.R., Rodriguez S. (2018). Cardiometabolic phenotypes and mitochondrial DNA copy number in two cohorts of UK women. Mitochondrion.

[86] Nicolson G.L. (2014). Mitochondrial dysfunction and chronic disease: Treatment with natural supplements. Integr. Med..

[87] Guha M., Avadhani N.G. (2013). Mitochondrial retrograde signaling at the crossroads of tumor bioenergetics, genetics and epigenetics. Mitochondrion.

[88] Cai N., Li Y., Chang S., Liang J., Lin C., Zhang X., Liang L., Hu J., Chan W., Kendler K.S. (2015). Genetic Control over mtDNA and Its Relationship to Major Depressive Disorder. Curr. Biol..

[89] Reznik E., Miller M.L., Şenbabaoğlu Y., Riaz N., Sarungbam J., Tickoo S.K., A Al-Ahmadie H., Lee W., E Seshan V., Hakimi A.A. (2016). Mitochondrial DNA copy number variation across human cancers. elife.

[90] Ravi S., Mitchell T., Kramer P.A., Chacko B., Darley-Usmar V.M. (2014). Mitochondria in monocytes and macrophages-implications for translational and basic research. Int. J. Biochem. Cell Biol..

[91] Martinez F.O., Sica A., Mantovani A., Locati M. (2008). Macrophage activation and polarization. Front. Biosci..

[92] Ashar F.N., Moes A., Moore A.Z., Grove M.L., Chaves P.H.M., Coresh J., Newman A.B., Matteini A.M., Bandeen-Roche K., Boerwinkle E. (2015). Association of mitochondrial DNA levels with frailty and all-cause mortality. J. Mol. Med..

[93] Ashar F.N., Zhang Y., Longchamps R.J., Lane J., Moes A., Grove M.L., Mychaleckyj J.C., Taylor K.D., Coresh J., Rotter J.I. (2017). Association of Mitochondrial DNA Copy Number With Cardiovascular Disease. JAMA Cardiol..

[94] Jeng J.-Y., Yeh T.-S., Lee J.-W., Lin S.-H., Fong T.-H., Hsieh R.-H. (2008). Maintenance of mitochondrial DNA copy number and expression are essential for preservation of mitochondrial function and cell growth. J. Cell. Biochem..

[95] Aggarwal N.T., Makielski J.C. (2013). Redox Control of Cardiac Excitability. Antioxid. Redox Signal..

[96] Yu E.P.K., Bennett M.R. (2014). Mitochondrial DNA damage and atherosclerosis. Trends Endocrinol. Metab..

[97] Berliner J.A., Heinecke J.W. (1996). The role of oxidized lipoproteins in atherogenesis. Free Radic. Biol. Med..

[98] Erusalimsky J.D. (2009). Vascular endothelial senescence: From mechanisms to pathophysiology. J. Appl. Physiol..

[99] Jain M., Rivera S., Monclus E.A., Synenki L., Zirk A., Eisenbart J., Feghali-Bostwick C., Mutlu G.M., Budinger G.R.S., Chandel N.S. (2013). Mitochondrial Reactive Oxygen Species Regulate Transforming Growth Factor-β Signaling. J. Biol. Chem..

[100] Liu M., Liu H., Dudley S.C. (2010). Reactive Oxygen Species Originating From Mitochondria Regulate the Cardiac Sodium Channel. Circ. Res..

[101] Lo I.C., Shih J.-M., Jiang M.J. (2005). Reactive oxygen species and ERK 1/2 mediate monocyte chemotactic protein-1-stimulated smooth muscle cell migration. J. Biomed. Sci..

[102] Sánchez-Santos A., Martínez-Hernández M.G., Contreras-Ramos A., Ortega-Camarillo C., Baiza-Gutman L.A. (2018). Hyperglycemia-induced mouse trophoblast spreading is mediated by reactive oxygen species. Mol. Reprod. Dev..

[103] Knez J., Winckelmans E., Plusquin M., Thijs L., Cauwenberghs N., Gu Y., Staessen J.A., Nawrot T.S., Kuznetsova T. (2015). Correlates of Peripheral Blood Mitochondrial DNA Content in a General Population. Am. J. Epidemiol..

[104] Tin A., Grams M.E., Ashar F.N., Lane J.A., Rosenberg A.Z., Grove M.L., Boerwinkle E., Selvin E., Coresh J., Pankratz N. (2016). Association between Mitochondrial DNA Copy Number in Peripheral Blood and Incident CKD in the Atherosclerosis Risk in Communities Study. J. Am. Soc. Nephrol..

[105] Zhang Y., Guallar E., Ashar F.N., Longchamps R.J., Castellani C.A., Lane J., Grove M.L., Coresh J., Sotoodehnia N., Ilkhanoff L. (2017). Association between mitochondrial DNA copy number and sudden cardiac death: Findings from the Atherosclerosis Risk in Communities study (ARIC). Eur. Heart J..

[106] Nymberg P., Memon A.A., Sundquist J., Sundquist K., Zöller B. (2021). Mitochondria-DNA copy-number and incident venous thromboembolism among middle-aged women: A population-based cohort study. J. Thromb. Thrombolysis.

[107] Di Leonardo A., Linke S.P., Clarkin K., Wahl G.M. (1994). DNA damage triggers a prolonged p53-dependent G1 arrest and long-term induction of Cip1 in normal human fibroblasts. Genes. Dev..

[108] Gire V., Dulic V. (2015). Senescence from G2 arrest, revisited. Cell Cycle.

[109] Muñoz-Espín D., Cañamero M., Maraver A., Gómez-López G., Contreras J., Murillo-Cuesta S., Rodríguez-Baeza A., Varela-Nieto I., Ruberte J., Collado M. (2013). Programmed cell senescence during mammalian embryonic development. Cell.

[110] Storer M., Mas A., Robert-Moreno À., Pecoraro M., Ortells M.C., Di Giacomo V., Yosef R., Pilpel N., Krizhanovsky V., Sharpe J. (2013). Senescence is a developmental mechanism that contributes to embryonic growth and patterning. Cell.

[111] Nguyen D., Jeon H.-M., Lee J. (2022). Tissue factor links inflammation, thrombosis, and senescence in COVID-19. Sci. Rep..

[112] Konieczyńska M., Natorska J., Undas A. (2023). Thrombosis and Aging: Fibrin Clot Properties and Oxidative Stress. Antioxid. Redox Signal..

[113] Poredos P., Jezovnik M.K. (2017). Endothelial Dysfunction and Venous Thrombosis. Angiology.

[114] Ocampo A., Reddy P., Martinez-Redondo P., Platero-Luengo A., Hatanaka F., Hishida T., Li M., Lam D., Kurita M., Beyret E. (2016). In Vivo Amelioration of Age-Associated Hallmarks by Partial Reprogramming. Cell.

[115] Atsem S., Reichenbach J., Potabattula R., Dittrich M., Nava C., Depienne C., Böhm L., Rost S., Schorsch M., Haaf T. (2016). Paternal age effects on sperm FOXK1 and KCNA7 methylation and transmission into the next generation. Hum. Mol. Genet..

[116] Potabattula R., Dittrich M., Böck J., Haertle L., Müller T., Hahn T., Schorsch M., El Hajj N., Haaf T. (2018). Allele-specific methylation of imprinted genes in fetal cord blood is influenced by cis-acting genetic variants and parental factors. Epigenomics.

[117] Edwards T.M., Myers J.P. (2007). Environmental exposures and gene regulation in disease etiology. Env. Health Perspect..

[118] Kulkarni H., Kos M.Z., Neary J., Dyer T.D., Kent J.W., Göring H.H., Cole S.A., Comuzzie A.G., Almasy L., Mahaney M.C. (2015). Novel epigenetic determinants of type 2 diabetes in Mexican-American families. Hum. Mol. Genet..

[119] Khare T., Pai S., Koncevicius K., Pal M., Kriukiene E., Liutkeviciute Z., Irimia M., Jia P., Ptak C., Xia M. (2012). 5-hmC in the brain is abundant in synaptic genes and shows differences at the exon-intron boundary. Nat. Struct. Mol. Biol..

[120] Moore L.D., Le T., Fan G. (2013). DNA Methylation and Its Basic Function. Neuropsychopharmacology.

[121] Chen Y., Breeze C.E., Zhen S., Beck S., Teschendorff A.E. (2016). Tissue-independent and tissue-specific patterns of DNA methylation alteration in cancer. Epigenetics Chromatin.

[122] Shemer R., Birger Y., Dean W.L., Reik W., Riggs A.D., Razin A. (1996). Dynamic methylation adjustment and counting as part of imprinting mechanisms. Proc. Natl. Acad. Sci. USA.

[123] Gartler S.M., Riggs A.D. (1983). Mammalian X-Chromosome Inactivation. Annu. Rev. Genet..

[124] Choi J., Lyons D.B., Kim M.Y., Moore J.D., Zilberman D. (2020). DNA Methylation and Histone H1 Jointly Repress Transposable Elements and Aberrant Intragenic Transcripts. Mol. Cell.

[125] Klutstein M., Nejman D., Greenfield R., Cedar H. (2016). DNA Methylation in Cancer and Aging. Cancer Res..

[126] Razin A., Riggs A.D. (1980). DNA methylation and gene function. Science.

[127] Unnikrishnan A., Hadad N., Masser D.R., Jackson J., Freeman W.M., Richardson A. (2018). Revisiting the genomic hypomethylation hypothesis of aging. Ann. N. Y. Acad. Sci..

[128] Hannum G., Guinney J., Zhao L., Zhang L., Hughes G., Sadda S., Klotzle B., Bibikova M., Fan J.-B., Gao Y. (2013). Genome-wide Methylation Profiles Reveal Quantitative Views of Human Aging Rates. Mol. Cell.

[129] Horvath S. (2013). DNA methylation age of human tissues and cell types. Genome Biol..

[130] Christiansen L., Lenart A., Tan Q., Vaupel J.W., Aviv A., McGue M., Christensen K. (2016). DNA methylation age is associated with mortality in a longitudinal Danish twin study. Aging Cell.

[131] Chen B.H., Marioni R.E., Colicino E., Peters M.J., Ward-Caviness C.K., Tsai P.C., Roetker N.S., Just A.C., Demerath E.W., Guan W. (2016). DNA methylation-based measures of biological age: Meta-analysis predicting time to death. Aging.

[132] Chambers J.C., Loh M., Lehne B., Drong A., Kriebel J., Motta V., Wahl S., Elliott H.R., Rota F., Scott W.R. (2015). Epigenome-wide association of DNA methylation markers in peripheral blood from Indian Asians and Europeans with incident type 2 diabetes: A nested case-control study. Lancet Diabetes Endocrinol..

[133] Ferrari L., Pavanello S., Bollati V. (2019). Molecular and epigenetic markers as promising tools to quantify the effect of occupational exposures and the risk of developing non-communicable diseases. Med. Lav..

[134] Wu C., Duan X., Wang X., Wang L. (2023). Advances in the role of epigenetics in homocysteine-related diseases. Epigenomics.

[135] Salybekov A.A., Hassanpour M. (2023). Unveiling the Genetic Footprint: Exploring Somatic Mutations in Peripheral Arterial Disease Progression. Biomedicines.

[136] Ward-Caviness C.K., Huffman J.E., Everett K., Germain M., van Dongen J., Hill W.D., Jhun M.A., Brody J.A., Ghanbari M., Du L. (2018). DNA methylation age is associated with an altered hemostatic profile in a multiethnic meta-analysis. Blood.

[137] Olivieri F., Prattichizzo F., Giuliani A., Matacchione G., Rippo M.R., Sabbatinelli J., Bonafè M. (2021). miR-21 and miR-146a: The microRNAs of inflammaging and age-related diseases. Ageing Res. Rev..

[138] Aïssi D., Dennis J., Ladouceur M., Truong V., Zwingerman N., Rocanin-Arjo A., Germain M., Paton T.A., Morange P.-E., Gagnon F. (2014). Genome-Wide Investigation of DNA Methylation Marks Associated with FV Leiden Mutation. PLoS ONE.

[139] Benincasa G., Costa D., Infante T., Lucchese R., Donatelli F., Napoli C. (2019). Interplay between genetics and epigenetics in modulating the risk of venous thromboembolism: A new challenge for personalized therapy. Thromb. Res..

[140] Zwingerman N., Kassam I., Truong V., Aïssi D., Dennis J., Wilson M.D., Wells P., Morange P.E., Trégouët D.-A., Gagnon F. (2015). Role of DNA methylation in candidate genes regions on tissue plasminogen activator levels. J. Thromb. Haemost..

[141] Rocañín-Arjó A., Dennis J., Suchon P., Aïssi D., Truong V., Trégouët D.-A., Gagnon F., Morange P.-E. (2015). Thrombin Generation Potential and Whole-Blood DNA methylation. Thromb. Res..

[142] Peters M.J., Joehanes R., Pilling L.C., Schurmann C., Conneely K.N., Powell J., Reinmaa E., Sutphin G.L., Zhernakova A., Schramm K. (2015). The transcriptional landscape of age in human peripheral blood. Nat. Commun..

[143] Pappireddi N., Martin L., Wühr M. (2019). A Review on Quantitative Multiplexed Proteomics. Chembiochem.

[144] Lundberg M., Eriksson A., Tran B., Assarsson E., Fredriksson S. (2011). Homogeneous antibody-based proximity extension assays provide sensitive and specific detection of low-abundant proteins in human blood. Nucleic Acids Res..

[145] Baird G.S., Nelson S.K., Keeney T.R., Stewart A., Williams S., Kraemer S., Peskind E.R., Montine T.J. (2012). Age-dependent changes in the cerebrospinal fluid proteome by slow off-rate modified aptamer array. Am. J. Pathol..

[146] Lu J., Huang Y., Wang Y., Li Y., Zhang Y., Wu J., Zhao F., Meng S., Yu X., Ma Q. (2012). Profiling plasma peptides for the identification of potential ageing biomarkers in Chinese Han adults. PLoS ONE.

[147] Menni C., Kiddle S.J., Mangino M., Viñuela A., Psatha M., Steves C., Sattlecker M., Buil A., Newhouse S., Nelson S. (2015). Circulating Proteomic Signatures of Chronological Age. J. Gerontol. A Biol. Sci. Med. Sci..

[148] Tanaka T., Basisty N., Fantoni G., Candia J., Moore A.Z., Biancotto A., Schilling B., Bandinelli S., Ferrucci L. (2020). Plasma proteomic biomarker signature of age predicts health and life span. Elife.

[149] Lehallier B., Gate D., Schaum N., Nanasi T., Lee S.E., Yousef H., Losada P.M., Berdnik D., Keller A., Verghese J. (2019). Undulating changes in human plasma proteome profiles across the lifespan. Nat. Med..

[150] Tanaka T., Biancotto A., Moaddel R., Moore A.Z., Gonzalez-Freire M., Aon M.A., Candia J., Zhang P., Cheung F., Fantoni G. (2018). Plasma proteomic signature of age in healthy humans. Aging Cell.

[151] Hipp M.S., Kasturi P., Hartl F.U. (2019). The proteostasis network and its decline in ageing. Nat. Rev. Mol. Cell Biol..

[152] Johnson A.A., Shokhirev M.N., Wyss-Coray T., Lehallier B. (2020). Systematic review and analysis of human proteomics aging studies unveils a novel proteomic aging clock and identifies key processes that change with age. Ageing Res. Rev..

[153] Menni C., Kastenmüller G., Petersen A.K., Bell J.T., Psatha M., Tsai P.-C., Gieger C., Schulz H., Erte I., John S. (2013). Metabolomic markers reveal novel pathways of ageing and early development in human populations. Int. J. Epidemiol..

[154] Rist M.J., Roth A., Frommherz L., Weinert C.H., Krüger R., Merz B., Bunzel D., Mack C., Egert B., Bub A. (2017). Metabolite patterns predicting sex and age in participants of the Karlsruhe Metabolomics and Nutrition (KarMeN) study. PLoS ONE.

[155] van den Akker E.B., Trompet S., Wolf J.J.B., Beekman M., Suchiman H.E.D., Deelen J., Asselbergs F.W., Boersma E., Cats D., Elders P.M. (2020). Metabolic Age Based on the BBMRI-NL ^1^H-NMR Metabolomics Repository as Biomarker of Age-related Disease. Circ. Genom. Precis. Med..

[156] Bingol K. (2018). Recent Advances in Targeted and Untargeted Metabolomics by NMR and MS/NMR Methods. High. Throughput.

[157] Gorrochategui E., Jaumot J., Lacorte S., Tauler R. (2016). Data analysis strategies for targeted and untargeted LC-MS metabolomic studies: Overview and workflow. TrAC Trends Anal. Chem..

[158] Lindström S., Wang L., Smith E.N., Gordon W., van Hylckama Vlieg A., de Andrade M., Brody J.A., Pattee J.W., Haessler J., Brumpton B.M. (2019). Genomic and transcriptomic association studies identify 16 novel susceptibility loci for venous thromboembolism. Blood.

[159] Thibord F., Klarin D., Brody J.A., Chen M.-H., Levin M.G., Chasman D.I., Goode E.L., Hveem K., Teder-Laving M., Martinez-Perez A. (2022). Cross-Ancestry Investigation of Venous Thromboembolism Genomic Predictors. Circulation.

[160] Bruzelius M., Iglesias M.J., Hong M.-G., Sanchez-Rivera L., Gyorgy B., Souto J.C., Frånberg M., Fredolini C., Strawbridge R.J., Holmström M. (2016). PDGFB, a new candidate plasma biomarker for venous thromboembolism: Results from the VEREMA affinity proteomics study. Blood.

[161] Yuan S., Xu F., Zhang H., Chen J., Ruan X., Li Y., Burgess S., Åkesson A., Li X., Gill D. (2024). Proteomic insights into modifiable risk of venous thromboembolism and cardiovascular comorbidities. J. Thromb. Haemost..

[162] Ten Cate V., Prochaska J.H., Schulz A., Koeck T., Robles A.P., Lenz M., Eggebrecht L., Rapp S., Panova-Noeva M., Ghofrani H.A. (2021). Protein expression profiling suggests relevance of noncanonical pathways in isolated pulmonary embolism. Blood.

[163] Jiang X., Zeleznik O.A., Lindström S., Lasky-Su J., Hagan K., Clish C.B., Eliassen A.H., Kraft P., Kabrhel C. (2018). Metabolites Associated With the Risk of Incident Venous Thromboembolism: A Metabolomic Analysis. J. Am. Heart Assoc..

[164] Febra C., Saraiva J., Vaz F., Macedo J., Al-Hroub H.M., Semreen M.H., Maio R., Gil V., Soares N., Penque D. (2024). Acute venous thromboembolism plasma and red blood cell metabolomic profiling reveals potential new early diagnostic biomarkers: Observational clinical study. J. Transl. Med..

